# (+)-Spectaline and Iso-6-Spectaline Induce a Possible Cross-Talk between Autophagy and Apoptosis in *Trypanosoma brucei rhodesiense*

**DOI:** 10.3390/tropicalmed4030098

**Published:** 2019-07-01

**Authors:** Kah Tee Lim, Chiann Ying Yeoh, Zafarina Zainuddin, Mohd. Ilham Adenan

**Affiliations:** 1 Malaysian Institute of Pharmaceuticals and Nutraceuticals (IPharm), National Institutes of Biotechnology Malaysia, Ministry of Science, Technology and Innovation, Blok 5-A, Halaman Bukit Gambir, Penang 11700, Malaysia; 2 Faculty of Medicine and Health Sciences, Universiti Putra Malaysia, UPM Serdang 43400, Selangor, Malaysia; 3 Analytical Biochemistry Research Centre (ABrC), Universiti Sains Malaysia, Penang 11800, Malaysia; 4 Faculty of Applied Sciences, Universiti Teknologi MARA, Shah Alam 40450, Selangor, Malaysia; 5 Universiti Teknologi MARA Pahang Campus, 26400 Bandar Tun Abdul Razak Jengka, Pahang, Malaysia

**Keywords:** (+)-spectaline, iso-6-spectaline, *Trypanosoma brucei rhodesiense*, autophagy, apoptosis, cross-talk

## Abstract

In our previous study, two known piperidine alkaloids (+)-spectaline (**1**) and iso-6-spectaline (**2**) were isolated from the leaves of *Senna spectabilis* and showed no toxic effect on L6 cells. In view of the potential use of piperidine alkaloids in *S. spectabilis* for the treatment of sleeping sickness, further investigation on the cell death actions of the parasite after treatment with compound **1** and **2** suggested that the treated parasites died by a process of autophagy based on the characteristic morphological alterations observed in intracellular *T. b. rhodesiense*. In search for apoptosis, interestingly, trypanosomes treated with high concentration of compound **1** and **2** after 72 h significantly induced an early apoptosis-like programmed cell death (PCD) such as phosphatidylserine (PS) exposure, loss of mitochondrial membrane potential and caspases activation. No DNA laddering discriminated late apoptosis event. Taken together, these findings demonstrated the potential of compound **1** and **2** as a natural chemotherapeutic capable of inducing a possible cross-talk between autophagy and apoptosis in *T. b. rhodesiense*.

## 1. Introduction

Human African Trypanosomiasis (HAT) also known as “sleeping sickness” is caused by two protozoan parasites, *Trypanosoma brucei rhodesiense* and *Trypanosoma brucei gambiense*. The trypanosomes are transmitted to humans by a bite of the infected *Glossina* spp. (tsetse fly) and multiply extracellularly in blood, lymph, and cerebrospinal fluid. The disease affects about 50 million people yearly in 36 sub-Saharan Africa, with an estimated incidence of about 30,000 cases per annum [1]. The disease has two distinct stages. In the early stage, trypanosomes reproduce in the hemolymphatic system of the patient, whereas in the second stage, trypanosomes cross the blood–brain barrier (BBB) to the central nervous system that results in a coma and finally, death of the patient if left untreated. Endemics are prevalent in rural regions where public health facilities needed for an effective treatment are absent. At present, there are only a few drugs registered for the treatment of HAT, such as suramin, pentamidine, melarsoprol, eflornithine, and nifurtimox. Except for eflornithine and nifurtimox, the other drugs were developed a half century ago [2,3]. Suramin and pentamidine are effective against the early stage of HAT, whereas the second stage of the disease can only be treated with melarsoprol and eflornithine. Drugs for the treatment of sleeping sickness are depending on the causative subspecies and their ability to cross the BBB. In addition, antigenic variation that protects the trypanosome to survive attack by the host’s immune response poses difficulties in developing vaccines for the treatment of the disease [4].

Since trypanosomes have been recognized as human pathogens, HAT has been considered one of the most devastating health and economic development problems in sub-Saharan Africa. Current treatment for HAT is very limited and not ideal due to toxicity issues and impractical administration regimes, a situation that poses significant challenges in poorly equipped areas with inadequate medical facilities and resources. There are no other treatments available if the strains of trypanosome have evolved resistance to the limited drugs currently used in chemotherapy [5]. In addition, except for eflornithine, the mechanisms of action of the current drugs remain poorly understood. Many pharmaceutical companies are unwilling to develop drugs for HAT because developing an effective drug are expensive and require capital, and knowing that their target markets are the poorer countries—Asia, Latin America, and Sub-Saharan Africa [6]. All of these factors highlight the need to screen and discover new drug leads with a high activity and low toxicity effect against HAT for future drug development. To overcome the problems of the current treatment for HAT, plants and their derived products could be an interesting source of lead compounds with a strong activity and low cytotoxicity effect. Several studies have demonstrated the anti-trypanosomal activity of plant natural products. The toxicity and possible anti-trypanosomal properties of the plants collected from the Malaysian forest have been evaluated through an *in vitro* approach for the identification of anti-trypanosomal agents in our high-throughput screening campaign in searching for potential anti-trypanosomal agents. Elucidation of molecular events of programmed cell death (PCD) in *T. b. rhodesiense* may lead to the discovery of new targets for future chemotherapeutic drug development.

**Figure 1:**
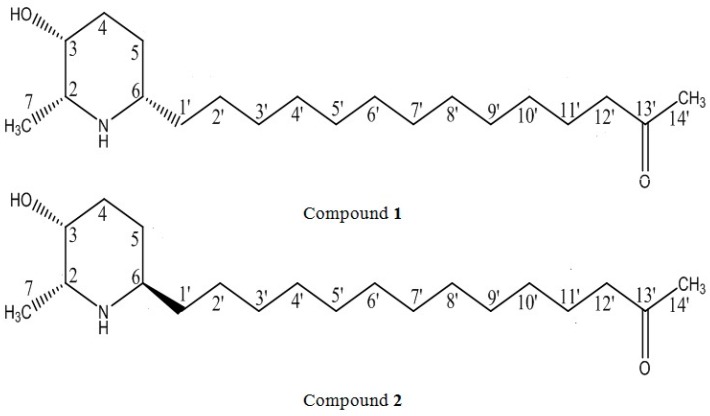
Chemical structure of piperidine alkaloids (+)-spectaline (**1**) and iso-6-spectaline (**2**) isolated from *S. spectabilis*.

In 2016, our group had isolated two piperidine alkaloids, (+)-spectaline (**1**) and iso-6-spectaline (**2**) (Figure 1) from the leaves of *Senna spectabilis* [7]. These two compounds inhibited growth of *T. b. rhodesiense* without toxic effect on mammalian L6 cells. Thus, it can be considered a potential candidate for HAT early drug discovery. Subsequently, the ultrastructural alterations in the trypanosome induced by these compounds leading to PCD were characterized using electron microscopy. These alterations include an unusual amount of trypanosome at the dividing state, wrinkling of the trypanosome surface, alterations in the kinetoplast, swelling of the mitochondria, and the formation of autophagosomes. These findings provide evidence of a possible autophagic cell death in *T. b. rhodesiense*. Furthermore, the formation of autophagic vacuoles and mitochondrial damages through monodansylcadaverine and MitoTracker Red labeling agreed with the electron microscopy data. Interestingly, trypanosomes treated with IC_90_ of compound **1** and **2** after 72 h exhibited an effect that significantly induced an early apoptosis-like PCD, which included phosphatidylserine (PS) exposure, loss of mitochondrial membrane potential, and caspases activation. Taken together, these findings demonstrated the potential of compound **1** and **2** as a natural chemotherapeutic. capable of inducing a possible cross-talk between autophagy and apoptosis in *T. b. rhodesiense*. This is the first report on the ultrastructural and cellular aspects of these two piperidine alkaloids on *T. b. rhodesiense*.

## 2. Materials and Methods

### 2.1. General Experimental Procedures

Trypanosomes were treated with **1** and **2** at IC_50_ (0.41 and 0.83 μM, respectively) and IC_90_ (0.71 and 1.21 μM, respectively) for 72 h based on the inhibitory effects previously determined by [7] and examined with cytochemical methods to determine the mode of cell death action in *T. b. rhodesiense*. The trypanosomes were grown in a T25 vented cap flask and incubated at 37 °C under a humidified atmosphere of 5% CO2 in air. Untreated trypanosomes served as a control. Treated and untreated trypanosomes were harvested by centrifugation at 2700 rpm for 10 min, washed in phosphate buffer saline (PBS) pH 7.4, and resuspended in respective solution as indicated. Data acquisition was immediately carried out on a flow cytometer (10,000 events were read) and analyzed with the CellQuest software. In a parallel experiment, cells stained with detection reagents were microscopically viewed on a confocal microscope. All the results were averaged from duplicates over three independent experiments.

### 2.2. Measurement of Mitochondrial Membrane Potential 

Mitochondrial damage of trypanosomes upon treatment with compound **1** and **2** were assessed by flow cytometry using a cell permeable dye MitoTracker Red CMXRos according to the manufacturer’s instructions (Invitrogen). MitoTracker Red was passively diffused via the plasma membrane of viable cells and accumulated in active mitochondria. Briefly, treated and untreated trypanosomes were harvested, washed, and resuspended in 100 μL of 100 nM MitoTracker Red. The trypanosome suspension was incubated for 15 min at 37 °C. After incubation, 400 μL of PBS was added to the suspension. Immediately, the trypanosome solutions were analyzed using flow cytometer.

### 2.3. Detection of PS Exposure

The annexin V-FITC staining was performed with ApoDetect™ Annexin V-FITC Kit as per manufacturer’s instructions (Invitrogen). Annexin V (a Ca^2+-^dependent phospholipid binding protein) has a high affinity for PS and is useful for characterizing apoptotic cells with externalized PS. Concurrent application of the DNA binding dye propidium iodide (PI) and analysis of the stained trypanosomes by flow cytometry was used to discriminate the late apoptotic or necrotic cells from the early apoptotic cells. Briefly, the treated and untreated samples were harvested, washed, and resuspended in 100 μL of 1× binding buffer. After that, 5 μL of annexin V-FITC and PI were added to the trypanosome suspensions and incubated for 15 min in the dark at room temperature. After incubation, 400 μL of 1× binding buffer was added to the suspensions. Immediately, data acquisition was carried out on a flow cytometer. Quantitative analysis of FITC fluorescence was done to show the PS exposure.

### 2.4. DNA Fragmentation Analysis

The DNA fragmentation analysis was performed with Quick Apoptotic DNA Ladder Detection Kit as per manufacturer’s instructions (Invitrogen). Treated and untreated trypanosomes were harvested, washed, and resuspended in 35 μL of Tris-EDTA lysis buffer. The mixture was incubated at 37 °C for 15 min in the presence of 5 μL of RNAase A. Next, 5 μL of proteinase K was added to the mixture and incubated at 60 °C for 30 min. After that, the solution was precipitated overnight at −20 °C with 5 μL of ammonium acetate solution and 100 μL of absolute alcohol. Following overnight incubation, the sample was centrifuged at 14,000 rpm for 10 min. The pellet was washed with 0.5 mL of 70% alcohol, air dried for 10 min, and resuspended in 50 μL DNA suspension buffer. DNA concentration was quantified at 260/280 nm on a spectrophotometer. DNA (10 μg/lane) was electrophoresed in 1.2% agarose gel containing 1× SYBR® Safe in 1× TAE buffer for 40 min at 70 V, visualized under UV light and photographed using gel documentation system.

### 2.5. Determination of Caspase-Like Protease Activity

Caspase-like protease activity was measured using caspase-3/7 green detection reagent according to the manufacturer’s instructions (Invitrogen). The reagent is a novel substrate for activated caspases-3 and -7. Briefly, treated and untreated samples were harvested, washed, and resuspended in 100 μL of 5 μM detection reagent. The trypanosome suspensions were incubated for 15 min at 37 °C. Immediately, the trypanosome solution was analyzed using flow cytometer.

### 2.6. Autophagy Assay

To confirm the formation of autophagy vacuoles, the effect of these compounds in the autophagic cell death was evaluated by monodansylcadaverine (MDC) labeling. MDC reagent (Sigma) is an autophagolysosome marker that is particularly taken up by autophagic cells and stains autophagosome structures. The MDC labeling was performed according to the protocol described by [8]. Briefly, the treated and untreated trypanosomes were harvested, washed, and resuspended in 100 μL of 0.05 mM MDC in PBS. After incubation for 15 min at 37 °C, trypanosomes were washed and lysed in 10 mM Tris-HCl pH 8.0 containing 1% SDS. Intracellular MDC was measured on a Clariostar® microplate reader (excitation wavelength of 380 nm and emission wavelength of 525 nm). To normalize the measurements to the number of trypanosomes, ethidium bromide was added to a final concentration of 2 μM and total DNA fluorescence was quantified on the Clariostar® microplate reader (excitation wavelength of 530 nm and emission wavelength of 590 nm). Results were expressed as specific activity (% respect to the control). The specific activity is the ratio of the MDC signal divided by the DNA concentration of the MDC assay. 

## 3. Results and Discussion

### 3.1. Measurement of Mitochondrial Membrane Potential

**Figure 2:**
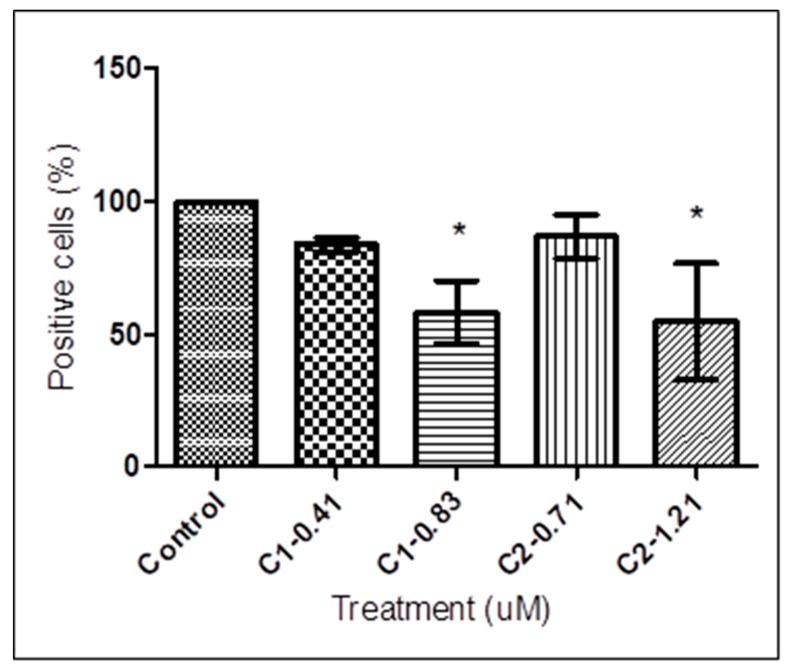
Quantitative analysis of mitochondrial membrane potential of *T***.** *b. rhodesiense*. Treated and untreated trypanosomes were exposed to **1** at 0.41 and 0.83 μM, and **2** at 0.71 and 1.21 μM for 72 h, stained with potentiometric probe MitoTracker Red (100 nM) for 15 min and analyzed by flow cytometry. Trypanosomes treated with 0.41 and 0.83 µM of **1,** and 0.71 and 1.21 µM of **2** resulted in the reduction of MitoTracker Red positive cells from 99.74 ± 0.11% (control) to 83.92 ± 2.68%, 58.31 ± 11.75%, 86.83 ± 8.19%, and 54.65 ± 22.04%, respectively. Data are presented as means ± SD from triplicates over three other independent experiments. The asterisks indicate significant differences between the treated and untreated groups calculated by one-way ANOVA with Tukey post-test (* *p* < 0.05). C, compound.

Alterations of the mitochondrial membrane potential (∆Ψm) upon treatment with **1** and **2** for *T. b. rhodesiense* were assessed by flow cytometry using cell permeable dye, MitoTracker Red CMXRos. MitoTracker Red is a red-fluorescent dye that stains active mitochondria in healthy cells, which passively diffuse across the plasma membrane and reside in mitochondria with an active membrane potential. Spectrofluorometric data presented in Figure 2 shows a marked decrease in fluorescence intensity (∆Ψm values), indicating depolarization of the membrane potential in cells following treatment with **1** at 0.41 and 0.83 μM and **2** at 0.71 and 1.21 μM. Trypanosomes treated with 0.41 and 0.83 µM of **1** resulted in a reduction of MitoTracker positive cells from 99.74 ± 0.11% (control) to 83.92 ± 2.68% and 58.31 ± 11.75%, respectively. On the other hand, treatment with **2** at 0.71 and 1.21 µM induced ∆Ψm reductions of 86.83 ± 8.19% and 54.65 ± 22.04%, respectively. As shown in Figure 2, there was virtually no difference between the control group and trypanosomes treated with 0.41 and 0.71 μM of **1** and **2**. In contrast to the trypanosomes treated with 0.41 and 0.71 μM, treatment with 0.83 and 1.21 μM of **1** and **2** exhibited significant depolarization of mitochondrial membrane potential (one-way ANOVA, *p* < 0.05) compared with untreated controls.

### 3.2. Detection of PS Exposure

**Figure 3:**
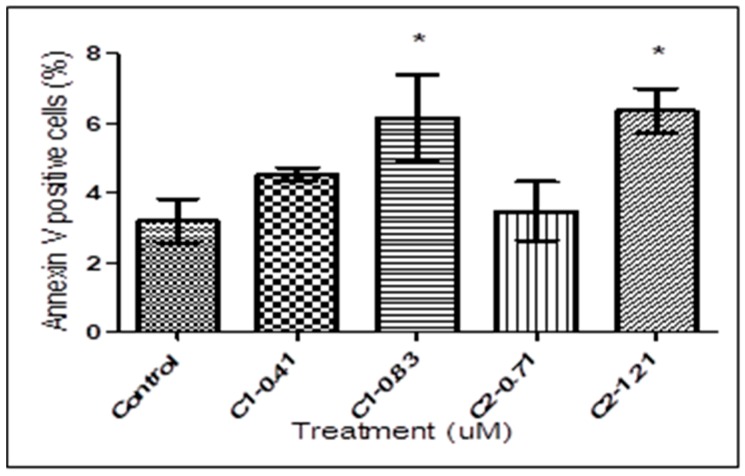
Fluorescence-activated cell sorting (FACS) analysis for PS exposure, measured by double staining with annexin V and PI in *T. b. rhodesiense*. Trypanosomes were treated with **1** at 0.41 and 0.83 μM and **2** at 0.71 and 1.21 μM for 72 h. An increased in annexin V-positive cells in treated trypanosomes (IC_90_) compared with untreated controls. The bars represent the means ± SD of three independent experiments. The asterisks indicate significant differences between the treated and untreated groups calculated by one-way ANOVA with Tukey post-test (* *p* < 0.05). UL, upper left; UR, upper right; LL, lower left; LR, lower right; C, compound.

During the early stage of apoptosis, PS was translocated to the outer layer of the plasma membrane and was exposed on the external surface of the cells. PS externalization was studied by annexin V binding of trypanosomes following bio-active compounds treatment. Quantitative analysis showed an increased in annexin V-positive cells in treated trypanosomes compared with untreated controls. As shown in Figure 3, there was virtually no difference between control group and trypanosomes treated with **1** at 0.41 and 0.83 μM and **2** at 0.71 and 1.21 μM. In contrast to the trypanosomes treated with 0.41 and 0.71 μM, treatment with 0.83 and 1.21 μM of **1** and **2** exhibited significant increases in the percentage of trypanosomes positive to annexin V (one-way ANOVA, *p* < 0.05) compared with untreated controls.

### 3.3. DNA Fragmentation Analysis

**Figure 4:**
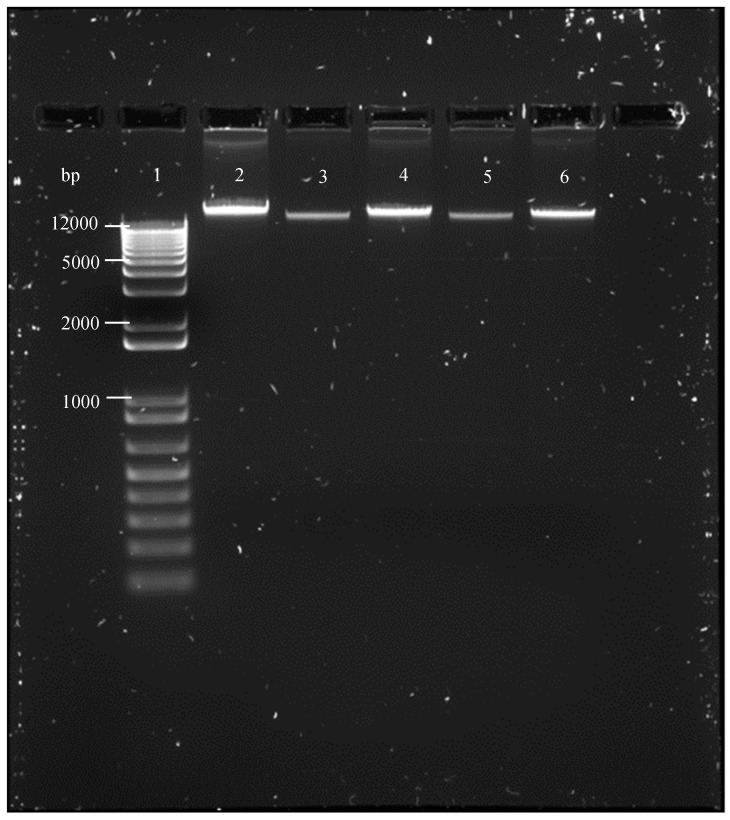
Analysis of DNA fragmentation in **1**- and **2**-treated *T. b. rhodesiense* for 72 h. Agarose gel represent the results of a typical experiment that produced similar results. Genomic DNA (10 µg/lane) from treated and untreated trypanosomes was resolved on a 1.2% agarose gel for 40 min at 70 V, then visualized under UV light. Lane 1, size marker 1 kb DNA ladder; lane 2, untreated trypanosomes; lane 3 and 4, DNA from trypanosomes treated with 0.41 and 0.83 µM of **1**; lane 5 and 6, DNA from trypanosomes treated with 0.71 and 1.21 µM of **2**. bp, base pair.

PCD through apoptotic pathways is identified by biochemical and morphological changes that result from nucleases and proteases activities. Together with the formation of apoptotic bodies and chromatin condensation, DNA laddering in oligonucleosomal fractions is one of the last steps in the apoptotic process. Oligonucleosomal DNA fragmentation analysis of *T. b. rhodesiense* was carried out following treatment of the trypanosomes with **1** at 0.41 and 0.83 μM and **2** at 0.71 and 1.21 μM. DNA laddering profiles presented in Figure 4 clearly demonstrate no fragmentation of the genomic DNA of the trypanosomes following treatment with **1** and **2** for 72 h.

### 3.4. Determination of Caspase-Like Protease Activity

**Figure 5:**
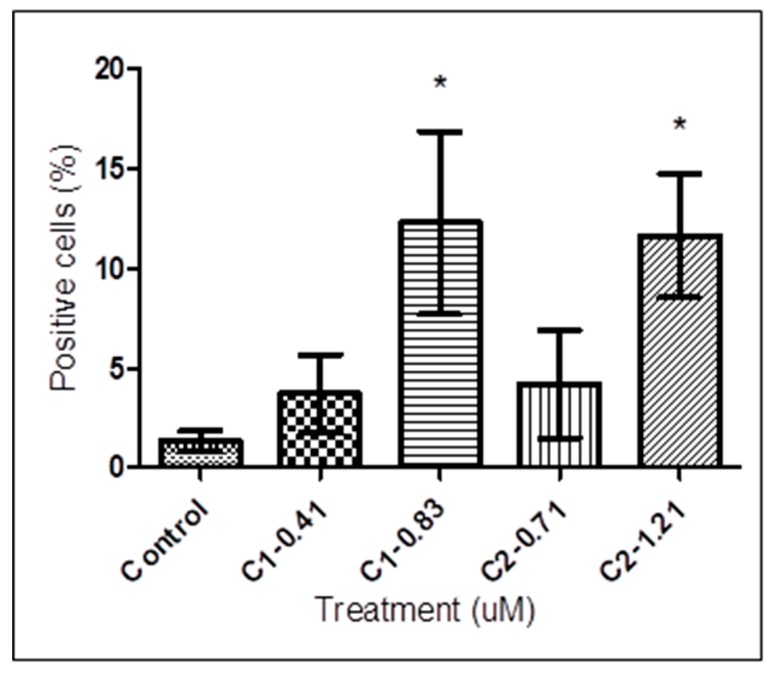
Quantitative analysis of caspase-like activity in *T. b. rhodesiense*. Treated and untreated trypanosomes were exposed to **1** at 0.41 and 0.83 μM, and **2** at 0.71 and 1.21 μM for 72 h, stained with caspase-3/7 green detection reagent (5 µM) for 15 min and analyzed by flow cytometry. Trypanosomes treated with 0.41 and 0.83 µM of **1,** and 0.71 and 1.21 µM of **2** resulted in an increment of caspase-3/7 positive cells from 1.35 ± 0.50% (control) to 3.70 ± 1.97%, 12.29 ± 4.59%, 4.20 ± 2.70%, and 11.64 ± 3.11%, respectively. Data presented as means ± SD from triplicates over three other independent experiments. The asterisks indicate significant differences between the treated and untreated groups calculated by one-way ANOVA with Tukey post-test (* *p* < 0.05). C, compound.

Caspases are activated to produce a controlled form of cell death. A fluorometric assay of caspase-3/7 was carried out using its specific cell-permeant substrate DEVD-peptide (Asp-Glu-Val-Asp) conjugated to a nucleic acid binding dye. This substrate is basically non-fluorescent because the DEVD-peptide inhibits the dye to bind to DNA. After the activation of caspase-3/7 in apoptotic cells, the substrate is cleaved by caspase-3/7 and the dye stains the nucleus to produce a bright green fluorescence. Trypanosomes treated with 0.41 and 0.83 µM of **1** resulted in an increment of caspase-3/7 positive cells from 1.35 ± 0.50% (control) to 3.70 ± 1.97% and 12.29 ± 4.59%, respectively. On the other hand, treatment with 0.71 and 1.21 µM of **2** induced increment of caspase-3/7 positive cells to 4.20 ± 2.70% and 11.64 ± 3.11%, respectively. As shown in Figure 5, there was virtually no difference between control group and trypanosomes treated with 0.41 and 0.71 µM of **1** and **2**. In contrast to the trypanosomes treated with 0.41 and 0.71 µM, treatment with 0.83 and 1.21 µM of **1** and **2** exhibited significant caspases-like activity (one-way ANOVA, *p* < 0.05) compared with untreated controls.

### 3.5. Autophagy Assay

**Figure 6:**
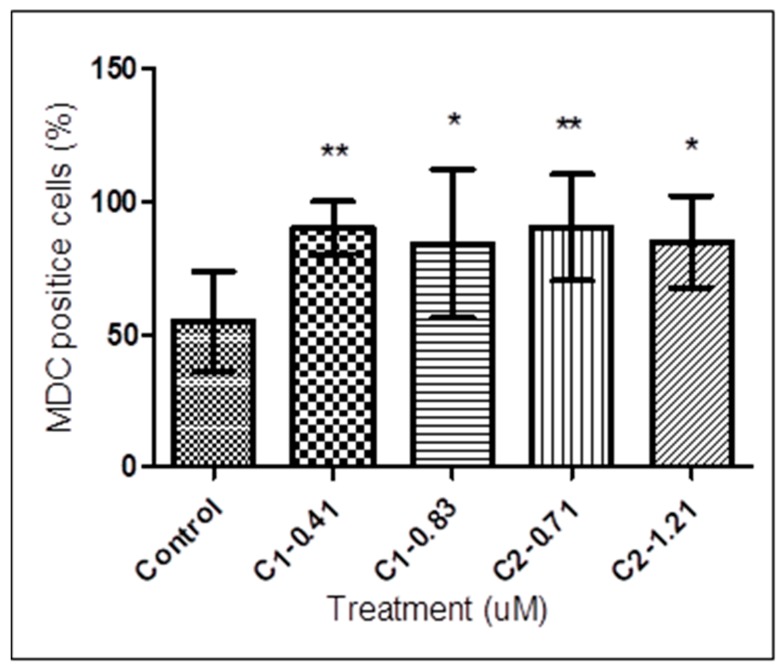
MDC labeling quantification of 1- and 2-treated *T. b. rhodesiense*. Treated and untreated trypanosomes were exposed to **1** at 0.41 and 0.83 μM and **2** at 0.71 and 1.21 μM for 72 h, stained with MDC (0.05 mM) for 10 min, normalized by staining with EtBr. The fluorescence signal was measured on a fluorometer. Trypanosomes treated with 0.41 and 0.83 µM of **1** resulted in increment of MDC positive cells from 56.21 ± 20.85% (control) to 90.40 ± 10.02% and 85.01 ± 27.94%, respectively. On the other hand, treatment with 0.71 and 1.21 µM of **2** induced increment of MDC positive cells to 90.29 ± 20.00% and 77.39 ± 17.25%, respectively. Data are presented as means ± SD from triplicates over three other independent experiments. The asterisks indicate significant differences between the treated and untreated groups, calculated by one-way ANOVA with Tukey post-test (* *p* < 0.05, ** *p* < 0.01). C, compound.

To confirm the formation of autophagic vacuoles in *T. b. rhodesiense* after 72 h treatment with **1** and **2**, the effect of these compounds in the autophagic cell death was evaluated. As shown in Figure 6, the percentage of autophagic cells positive to MDC staining was increased from 56.21 ± 20.85% in untreated cells (control) to 90.40 ± 10.02%, 85.01 ± 27.94%, 90.29 ± 20.00%, and 77.39 ± 17.26% following treatment with 0.41 and 0.83 µM of **1** and 0.71 and 1.21 µM of **2**, respectively. Using MDC staining, a significant staining difference between control cells and treated trypanosomes was observed by one-way ANOVA analysis followed by Tukey’s multiple comparison tests.

[9] have found that not all alkaloids induced PCD. In addition, the literature reports most tropane, quinolizidine, piperine, pyridine, purine, steroidal, and diterpene alkaloids were inactive up to a concentration of 100 μM. Therefore, the piperidine alkaloids **1** and **2** in this study represent interesting lead structures for potential anti-trypanosomal drugs. In our previous study, the observed changes are too small to provide evidence for apoptosis- or necrosis- like PCD suggesting an autophagic cell death due to compound **1** and **2** treatments, as there were no late apoptotic features shown by the parasites which include membrane blebbing, DNA laddering, cellular shrinkage, and the formation of apoptotic bodies [7]. Interestingly, compound **1** and **2** were also found to induce early apoptosis-like PCD in *T. b. rhodesiense*. Externalization of PS in the outer surface of the plasma membrane and caspases-like activity led to a dose-dependent increase of early apoptotic-like PCD in trypanosomes treated with **1** and **2** were evidently stronger at 0.83 and 1.21 µM. Taken together, these findings demonstrated the potential of compound **1** and **2** as a natural chemotherapeutic capable of inducing a possible cross-talk between autophagy and apoptosis in *T. b. rhodesiense*. Fungi, plant, and protozoa express metacaspases because they lack caspases. Although metacaspases have some similarity to the multicellular caspases, literature data do not give any evidence regarding the direct involvement of *T. brucei* metacaspases in apoptosis [10]. However, in a recent study, [11] proposed that metacaspases in kinetoplastid parasites such as *L. major* plays a vital role in the apoptosis and in the autophagy pathway. Therefore, in the context of these recent findings on trypanosomes, the caspase-like activity reported earlier needs to be examined. It is not uncommon for the cross-talk between autophagy and apoptosis to happen in kinetoplastids such as *T. cruzi* [12] and *L. major* [11]. To the best of our knowledge, this is the first report on the cellular aspects of these compounds in *T. b. rhodesiense*. 

## 4. Conclusions

In view of the potential use of piperidine alkaloids in *S. spectabilis* for the treatment of sleeping sickness, further investigation into the cell death actions of the parasite after treatment with compound **1** and **2** suggested that the treated parasites died by a process of autophagy based on the characteristic morphological alterations observed in intracellular *T. b. rhodesiense*. In the search for apoptosis, interestingly, the trypanosomes treated with a high concentration (IC_90_) of compound **1** and **2** after 72 h significantly induced an early apoptosis-like PCD. No DNA laddering discriminated against a late apoptosis event. Taken together, these findings demonstrated the potential of compound **1** and **2** as a natural chemotherapeutic capable of inducing a possible cross-talk between autophagy and apoptosis in *T. b. rhodesiense*. Further clarification of the molecular events related to the cross-talk for PCD that was induced in *T. b. rhodesiense* by compound **1** and **2** is of great interest as the full elucidation of the mechanism of cell death would help to identify new possible targets for the chemotherapeutic intervention in trypanosomiasis. Due to the promising results obtained, in vivo studies are suggested to confirm the therapeutic potential of both active piperidine alkaloids. Besides that, continued investigation of cross-talk between autophagy and apoptosis is necessary to elucidate the mechanisms controlling the balance between survival and death in stress response conditions caused by chemotherapeutic natural products. Understanding of mechanisms that regulate the cross-talk for PCD is important for discovery of therapeutic tools in combating the disease.
